# Increased Risk of Vertebral Fractures in Patients With Mild Autonomous Cortisol Secretion

**DOI:** 10.1210/clinem/dgad560

**Published:** 2023-09-21

**Authors:** Vittoria Favero, Cristina Eller-Vainicher, Valentina Morelli, Elisa Cairoli, Antonio Stefano Salcuni, Alfredo Scillitani, Sabrina Corbetta, Silvia Della Casa, Giovanna Muscogiuri, Luca Persani, Iacopo Chiodini

**Affiliations:** Department of Medical Biotechnology and Translational Medicine, University of Milan, 20100 Milan, Italy; Unit of Endocrinology, Fondazione IRCCS Cà Granda-Ospedale Maggiore Policlinico, 20100 Milan, Italy; Endocrinology Department of Endocrine and Metabolic Diseases, IRCCS Istituto Auxologico Italiano, 20100 Milan, Italy; Endocrinology Department of Endocrine and Metabolic Diseases, IRCCS Istituto Auxologico Italiano, 20100 Milan, Italy; Unit of Endocrinology and Metabolism, University-Hospital S. Maria Della Misericordia, 33100 Udine, Italy; Unit of Endocrinology, “Casa Sollievo della Sofferenza,” Hospital, IRCCS, San Giovanni Rotondo, 71013 Foggia, Italy; Endocrinology Department of Endocrine and Metabolic Diseases, IRCCS Istituto Auxologico Italiano, 20100 Milan, Italy; Department of Biomedical, Surgical and Dental Sciences, University of Milan, 20100 Milan, Italy; Department of Medical and Surgical Sciences, Fondazione Policlinico Universitario A. Gemelli, 00100 Rome, Italy; Dipartimento di Medicina Clinica e Chirurgia, Unità di Endocrinologia, Andrologia e Diabetologia - University of Naples “Federico II”, 80131 Naples, Italy; UNESCO Chair “Education for Health and Sustainable Development”, University of Naples “Federico II”, 80131 Naples, Italy; Department of Medical Biotechnology and Translational Medicine, University of Milan, 20100 Milan, Italy; Endocrinology Department of Endocrine and Metabolic Diseases, IRCCS Istituto Auxologico Italiano, 20100 Milan, Italy; Department of Medical Biotechnology and Translational Medicine, University of Milan, 20100 Milan, Italy; Unit of Endocrinology, ASST Grande Ospedale Metropolitano Niguarda, 20162 Milan, Italy

**Keywords:** mild hypercortisolism, fragility fracture, adrenal incidentaloma

## Abstract

**Context:**

The risk of vertebral fractures (VFx) in patients with adrenal incidentalomas (AI) and mild autonomous cortisol secretion (MACS) is debated.

**Objective:**

To evaluate the VFx prevalence and incidence in patients with AI and MACS.

**Methods:**

This cross-sectional and longitudinal study using retrospective data from 4 Italian endocrinology units included 444 patients (cross-sectional arm) and 126 patients (longitudinal arm, 24.9 ± 5.3 months follow-up) to evaluate prevalent and incident VFx, respectively, in patients with MACS (MACS-yes) and without MACS (MACS-no). The main outcome measures were serum cortisol after a 1-mg dexamethasone test (F-1mgDST), bone mineral density (BMD) by dual-energy x-ray absorptiometry at spine (LS) and femur (FN), and VFx presence by x-ray.

**Results:**

Cross-sectional arm: 214 and 230 patients were MACS-yes and MACS-no, respectively, based on F-1mgDST >1.8 µg/dL (50 nmol/L). Patients with MACS had higher VFx prevalence (62.6%) than those without MACS (22.9%, *P* < .001); MACS was associated with prevalent VFx (odds ratio, 5.203; 95% CI, 3.361-8.055; *P* < .001; relative risk [RR] 2.07), regardless of age, body mass index, gender distribution, LS-BMD, and presence of type 2 diabetes mellitus (T2D). Longitudinal arm: 66 and 60 patients were MACS-no and MACS-yes, respectively. Patients without MACS showed higher number of incident VFx (36.4%) than patients without MACS (10.0%, *P* < .001); MACS was associated with the presence of an incident VFx (RR 4.561; 95% CI, 1.600-13.003; *P* = .005) regardless of age, LS-BMD, gender distribution, presence of prevalent VFx, and T2D. Results were confirmed in women and men when separately evaluated.

**Conclusion:**

Women and men with AI and MACS are at higher risk of VFx.

The term *mild hypercortisolism* (also known as *hidden hypercortisolism* or *subclinical hypercortisolism* or *subclinical Cushing syndrome*) defines a condition of biochemical hypercortisolism in the absence of the classical clinical features of cortisol excess (1, 2). The importance of this disorder is related to the fact that: (i) it is associated with the same cardiometabolic consequences of the clinically overt cortisol excess (ie, Cushing syndrome) (3, 4); and (ii) unlike Cushing's syndrome, it is not a rare disease, being present in up to 50% of patients with adrenal incidentalomas (AI), which, in turn, are found in up to 7% of individuals older than 60 years (5). Among the possible consequences of mild hypercortisolism, several reports suggest that patients with AI and with mild hypercortisolism have an increased prevalence and incidence of fragility fractures and, of importance, that the recovery from mild hypercortisolism meaningfully decreases the fracture risk (6, 7).

Very recently, the European Society of Endocrinology (ESE) in collaboration with the European Network for the Study of Adrenal Tumors (ENSAT) released the new guidelines for diagnosis of mild hypercortisolism, now defined by the term *mild autonomous cortisol secretion* (MACS) (8, 9). The new criteria suggest that diagnosis of the MACS condition in patients with AI should be based on the presence of serum cortisol after a 1-mg dexamethasone (DST) test (F-1mgDST) result above 50 nmol/L or 1.8 µg/dL, in the absence of the classical signs or symptoms of overt Cushing syndrome. Since most of the available data on the risk of fragility fractures, mainly vertebral fractures (VFx), in patients with mild hypercortisolism were obtained from studies using different criteria for defining MACS, the recent ESE-ENSAT guidelines consider that the association between MACS and osteoporosis is not yet well established. However, the same guidelines suggest screening patients with AI and with MACS for VFx (8).

To date, the only available study on the risk of fractures in MACS suggests that postmenopausal women with AI and MACS have an increased prevalence of fragility fractures (10). There are no available data on the risk of incident fragility fractures in AI patients with MACS. Therefore, the aim of the present study was to evaluate the prevalence and incidence of vertebral fractures in a cohort of patients with AI and with or without MACS.

## Methods

### Design of the Study

In the present study, we re-analyzed the dataset of a previous retrospective study, designed for evaluating if the indexes of hypothalamic-pituitary-adrenal (HPA) axis activity could be used for identifying the AI patients at risk of VFx (11).

In a cross-sectional arm of the study, we first analyzed data from 444 patients with AI (271 women, 173 men) in order to compare the VFx prevalence in patients with AI and with MACS (MACS-yes) and in patients with AI but without MACS (MACS-no). Subsequently, in the longitudinal arm of the study, we evaluated in a group of 126 patients with AI, of whom follow-up data (at least 24 months) were available, the risk of VFx in patients with MACS as compared to patients without MACS.

### Subjects

The enrollment criteria have been already described elsewhere (11). Briefly, the subjects included in the cross-sectional arm of the study were enrolled from January 1997 to June 2013 in 4 referral Italian Hospitals. The longitudinal arm included patients with AI enrolled in the same centers from January 2005 to June 2013.

By definition, all AI were incidentally found by computed tomography (CT) scan, ultrasonography, or magnetic resonance imaging performed for unrelated diseases. Adrenal lesions found by ultrasound were subsequently confirmed with unenhanced CT. At CT scan, all adrenal tumors were larger than 1 cm in size and with well-shaped features consistent with the diagnosis of adrenocortical adenoma (ie, hypodense, homogeneous, and with Hounsfield units below 10). No subject had evidence of metastatic diseases with no biochemical evidence of aldosterone co-secretion.

The exclusion criteria were: (i) clinically evident cortisol excess (ie, presence of striae rubrae, moon facies, buffalo hump, proximal muscle weakness, and skin atrophy); (ii) history of hypogonadism (in premenopausal women less than 6 menstrual cycles/year, in men with testosterone levels less than 300 ng/dL) and/or presence of chronic kidney and hepatic disease, of bowel diseases, of eating disorder, of thyrotoxicosis, of rheumatologic or hematological diseases and of alcoholism; (iii) use of drugs influencing cortisol metabolism or secretion or dexamethasone metabolism; and (iv) the presence of bilateral adrenal masses (12).

Morning plasma adrenocorticotropin (ACTH) levels (normal values 10-55 pg/mL, 2.2-12 pmol/L), 24-hour urinary cortisol (UFC) levels (normal values, 10-70 µg/24 hours, 28-193 nmol/24 hours) and F-1mgDST were measured in all patients. In all patients with F-1mgDST >1.8 µg/dL (50 nmol/liter) and ACTH levels at >10 pg/mL (2.2 pmol/liter), an ACTH-dependent subclinical hypercortisolism was ruled out by a test with a corticotroph-releasing-hormone stimulation (13).

Bone mineral density (BMD) was measured in all patients by dual-energy x-ray absorptiometry (Hologic Discovery, software version 13.3:3) at both femoral neck (FN, precision 1.8%) and lumbar spine (LS, precision 1.0%) and reported as SD units (Z-score). In all patients a conventional dorsal-lumbar (T4-L4) spinal radiograph in lateral and anteroposterior projection was performed and reviewed by a trained radiologist, who was blinded to BMD and hormonal data. The semiquantitative visual assessment was used to diagnose prevalent and incident VFx (14). The presence of *osteoporosis* is defined by a BMD T-score < −2.5 at any skeletal site and/or the presence of a fragility fracture (15). As our sample included post- and premenopausal women and men younger than 50, the term *low BMD* was used to define individuals with T-score at any site ≤ −2.5 for postmenopausal women and men older than 50 or with Z-score at any site < −2.0 for premenopausal women and men younger than 50 years of age (16).

Finally, in all patients the body mass index (BMI) was calculated, the presence of type 2 diabetes mellitus (T2D) was evaluated using the World Health Organization criteria (17) and the presence of hypertension was defined as the presence of systolic blood pressure >140 mmHg.

All subjects signed the informed consent before entering the study. The protocol was approved by the Ethics Committees of the participating centers.

### Analytical Methods

In accordance with the Italian guidelines (18), all patients with hypovitaminosis D were supplemented with cholecalciferol per os to achieve normal vitamin D levels (ie, above 30 ng/mL, 75 nmol/L). All patients with insufficient dietary calcium intake (ie, less than 1000 mg/d) were supplemented with oral calcium carbonate or calcium citrate, as appropriate. Plasma ACTH levels were measured by immunoradiometric assay (BRAHMS Diagnostica GmbH) and reported as the mean of 3 determinations at 20-minute intervals. At baseline, in all patients we measured serum cortisol and UFC levels (after dichloromethane extraction) immunofluorimetrically by TDxFLx kits (Abbott GmbH Diagnostika). The intra- and interassay coefficients of variation were <15% for ACTH and <10% for all other assays. In both cross-sectional and longitudinal arms, the reported HPA axis parameters were those determined at baseline.

### Statistical Analysis

Statistical analysis was performed by SPSS, version 28.0 (Statistics for Data Analysis). The results were expressed as mean ± SD or as median (range) for not normally distributed variables. The normality of distribution was tested by Kolmogorov-Smirnov test. The comparison of continuous variables between patients with and without MACS was performed using the Student *t* test or the Mann-Whitney U test as appropriate. Categorical variables were compared by χ2 test or Fisher exact test, as appropriate.

The multivariate analysis assessed the independent associations between the presence of prevalent VFx (in the cross-sectional arm) or of incident VFx (in the longitudinal arm) after adjusting for the variables that resulted to be different between patients with MACS and those without MACS and for the possible influencing factors, such as age, BMI, gender, BMD, and presence of T2D, which is known to possibly increase VFx risk regardless for BMD (19). In the multivariate analysis, the presence of prevalent VFx was included among the covariates possibly predicting the presence of incident VFx in the longitudinal arm, since it is known that a previous fragility fracture increases the risk of subsequent fractures (20). The results have been expressed as adjusted odds ratio (aOR) and adjusted relative risk (aRR) (21). The adjusted hazard ratio (aHR) has been calculated by Cox regression.

## Results

### Cross-Sectional Arm: All Subjects

The demographic, hormonal, clinical, and radiological data are detailed in Table 1. Gender distribution, BMI, prevalence of T2D, and proportion of premenopausal status for women were comparable between the 2 groups. According to clinical and radiological characteristics, patients with MACS were older and showed higher adenoma size. As expected, patients with MACS showed higher F-1mgDST and UFC levels, lower ACTH levels, and higher prevalence of low ACTH levels (ie < 10 pg/mL, 2.2 pmol/L) than patients without MACS. In patients with MACS, the prevalence of osteoporosis was higher while LS-BMD and FN-BMD were lower than in those without MACS. The prevalence of all VFx (asymptomatic and symptomatic) and that of symptomatic VFx were both higher in patients with MACS (symptomatic VFx 7.0%) than in patients without MACS (symptomatic VFx 2.3%, *P* = .022).

**Table 1.** Clinical and biochemical characteristics of all patients with adrenal incidentalomas and of patients with or without mild autonomous cortisol secretion

	Total (n = 444)	MACS-no (n = 214)	MACS-yes (n = 230)	*P* value
Age (years)	61.8 ± 11.5 (21-89)	59.9 ± 13.0 (21-89)	63.6 ± 9.5 (24-83)	**<**.**001**
Women	271 (61.0)	134 (62.6)	137 (59.6)	.559
BMI (kg/m^2^)	29.2 ± 4.8 (19.5-40.9)	29.7 ± 5.0 (19.5-40.9)	28.7 ± 4.6 (19.5-40.9)	.27
Premenopausal women	31 (11.4)	21 (15.7)	10 (7.3)	.**03**
F-1mgDST (µg/dL)	2.39 ± 1.89 (0.50-12.0)	1.14 ± 0.38 (0.50 –1.8)	3.60 ± 2.00 (1.84-12.00)	**<**.**001**
UFC (µg/24 hours)	54.65 ± 32.07 (10.0-175.3)	50.42 ± 27.73 (10.0-169.1)	58.58 ± 35.24 (10.0-175.3)	.**007**
ACTH (pg/mL)	12.99 ± 9.39 (1.6-48.3)	15.89 ± 9.42 (2.8-48.3)	10.29 ± 6.21 (1.6-48.0)	**<**.**001**
Low ACTH	234 (52.7)	56 (26.2)	154 (67.0)	**<**.**001**
Tumor size (cm)	2.58 ± 1.1 (0.8-8.0)	2.15 ± 0.87 (0.8-6.0)	2.98 ± 1.16 (0.8-8.0)	**<**.**001**
Type 2 diabetes mellitus	73 (16.4)	32 (15)	41 (17.8)	.246
Osteoporosis	224 (50.5)	77 (36)	147 (63.9)	**<**.**001**
Prevalent VFx	193 (43.5)	49 (22.9)	144 (62.6)	**<0**.**001**
LS-BMD (Z-score)	0.10 (−4.50-3.61)	0.20 (−3.60-3.61)	−0.10 (−4.50-3.61)	.**005**
FN-BMD (Z-score)	−0.02 ± 1.07 (−2.80-5.33)	0.13 ± 1.12 (−2.80-5.33)	−0.16 ± 1.01 (−2.50-2.70)	.**004**

Categorical variables are reported as absolute number with percentage in parentheses. Continuous variables are reported as mean ± SD or median with range in parentheses. Statistically significant comparisons are in bold.

Abbreviations: ACTH, adrenocorticotrophic hormone; BMD, bone mineral density; BMI, body mass index; F-1mgDST: 1-mg overnight dexamethasone suppression test; FN, femoral neck; Low ACTH, ACTH levels <10.0 pg/mL (2.2 pmol/L); LS, lumbar spine; MACS, mild autonomous cortisol secretion; MACS-no, patients without mild autonomous cortisol secretion (F-1mgDST ≤1.8 µg/dL µg/dL, 50 nmol/L); MACS-yes, patients with mild autonomous cortisol secretion (F-1mgDST >1.8 µg/dL µg/dL, 50 nmol/L); Osteoporosis, BMD T-score < −2.5 at any skeletal site and/or the presence of a fragility fracture; UFC, urinary free cortisol.

**Figure 1.**
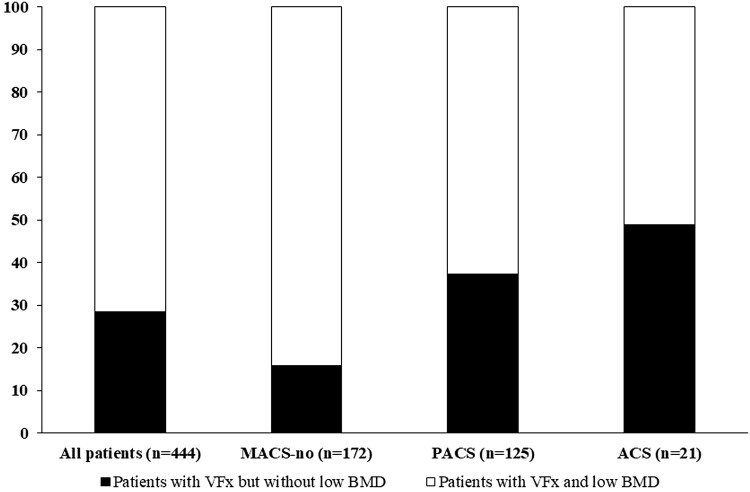
Prevalence of vertebral fractures at baseline in relation with the presence of low bone mineral density in all subjects with adrenal incidentalomas (AI), in patients without MACS, in patients with PACS, and in patients with autonomous cortisol secretion (ACS). VFx prevalence is higher in ACS (70.7%) compared with PACS and MACS-no patients (59.3% and 22.5%, respectively) and in PACS patients as compared with MACS-no patients (*P* < 0.001 for all comparisons). The percentage of individuals without low BMD but with prevalent VFx significantly increased with increasing cortisol secretion (MACS-no 15.7%, PACS 37.2%, ACS 48.4%, *P* < 0.001). Abbreviations: ACS, patients with autonomous cortisol secretion (F-1mgDST >5.0 µg/dL, 138 nmol/L) (8); MACS-no, patients without mild autonomous cortisol secretion (F-1mgDST ≤1.8 µg/dL µg/dL, 50 nmol/L); PACS, patients with possible autonomous cortisol secretion (F-1mgDST between 1.8 µg/dL and 5.0 µg/dL, 50-138 nmol/L).

Based on F-1mgDST levels, we subdivided patients with MACS into those with possible autonomous cortisol secretion (PACS, with F-1mgDST >1.8 µg/dL and ≤5.0 µg/dL, 50-138 nmol/L, n = 199) and those with autonomous cortisol secretion (ACS, with F-1mgDST >5.0 µg/dL, 138 nmol/L, n = 41), as defined in the previous European Society of Endocrinology Guidelines (9). We found a statistically significant difference between the prevalence of VFx in patients with ACS (70.7%) compared to those with PACS and to those without MACS (59.3% and 22.5%, respectively) and in patients with PACS patients than in patients without MACS (*P* < .001 for all comparisons). Moreover, the percentage of individuals without low BMD but with prevalent VFx significantly increased with increasing cortisol secretion (MACS-no 15.7%, PACS 37.2%, ACS 48.4%, *P* < .001) (Fig. 1). Finally, the presence of a prevalent VFx was associated with the presence of MACS-yes (aRR 2.07), BMD, and age, regardless of T2D presence, BMI, and gender distribution (Table 2).

**Table 2.** Independent associations between the presence of a prevalent vertebral fragility fracture and MACS, age, body mass index, gender, type 2 diabetes mellitus, and lumbar spine bone mineral density

	aOR	95% CI	*P* value
MACS (presence vs absence)	5.203	3.361-8.055	**<**.**001**
Age (1-y increase)	1.049	1.026 - 1.073	**<**.**001**
BMI (1 kg/m^2^ increase)	1.030	0.984 - 1.079	.201
Gender (women)	1.114	0.716 - 1.732	.632
LS-BMD (1 Z-score decrease)	1.395	1.182-1.645	**<**.**001**
Type 2 diabetes mellitus (presence vs absence)	1.391	0.781-2.481	.263

Statistically significant associations are in bold.

Abbreviations: aOR, odds ratio adjusted for the variables included in the model; BMD, bone mineral density; BMI, body mass index; LS, lumbar spine; MACS, mild autonomous cortisol secretion; MACS-yes, patients with mild autonomous cortisol secretion; MACS-no, patients without mild autonomous cortisol secretion; UFC, urinary free cortisol.

We compared patients with low ACTH levels (ie, < 10 pg/mL, 2.2 pmol/L) with those without low ACTH levels. The former subjects showed higher frequency of VFx (57.6%) than the latter subjects (30.8%, *P* < .001). Finally, the presence of a prevalent VFx was significantly associated with the presence of low ACTH levels even after adjusting for age, T2D presence, BMI, and gender distribution (data not shown).

### Longitudinal Arm: All Subjects

Duration of follow-up, BMI, BMD at both LS and FN, and prevalence of T2D were comparable between patients with MACS and those without MACS, while size of the adenoma, prevalence of women, and prevalence of low ACTH levels was higher and ACTH levels were lower in the former than in the latter group (Table 3). Moreover, the number of all incident VFx was higher in patients with MACS than in those without MACS, with the difference in symptomatic VFx trending toward significance (1.7% vs 9.1%, respectively, *P* = .069). The results did not change even after the exclusion of 4 patients with ACS (data not shown). The occurrence of an incident VFx was independently predicted by the presence of MACS-yes but not by age, presence of prevalent VFx, presence of T2D, gender distribution, duration of follow-up, and LS-BMD (Table 4). The adjusted hazard ratio of MACS-yes for predicting incident VFx over time after adjusting for the same variables was 2.884 (95% CI, 1.108-7.507; *P* = .03). Finally, the percentage of individuals without low BMD but with incident VFx was significantly higher in patients with MACS (25.8%) than in patients without MACS (8.3%, *P* = .017).

**Table 3.** Clinical and biochemical characteristics of all patients with adrenal incidentalomas and of patients with or without mild autonomous cortisol secretion included in the longitudinal arm

	Total (n = 126)	MACS-no (n = 60)	MACS-yes (n = 66)	*P* value
Age (years)	63.5 ± 9.5 (27 – 83)	61.5 ± 10.6 (27 – 80)	65.5 ± 8.0 (40 – 83)	.**023**
Women	80 (63.5)	35 (53.0)	45 (75.0)	.**016**
Premenopausal women	11 (13.8)	6 (13.3)	5 (14.3)	.920
BMI (kg/m^2^)	27.0 ± 4.3 (19.5 – 40.9)	27.2 ± 4.5 (20.1 – 40.9)	26.7 ± 4.1 (19.5 – 37.0)	.531
Follow-up (months)	24.9 ± 5.3 (24 – 72)	24.0 ± 0.0 (24 – 24)	25.6 ± 7.3 (24 – 72)	.083
F-1mgDST (µg/dL)	2.20 ± 1.20 (0.50 – 7.50)	1.20 ± 0.40 (0.50 – 1.80)	3.00 ± 1.10 (1.84 – 7.50)	**<**.**001**
UFC (µg/24 hours)	49.5 ± 31.1 (10.0 – 175.3)	43.8 ± 24.5 (10.0 – 119.4)	54.7 ± 35.5 (10.0 – 175.3)	.**050**
ACTH (pg/mL)	13.2 ± 8.2 (3.0 – 35.0)	16.1 ± 8.9 (5.0 – 35.0)	10.5 ± 6.5 (3.0 – 35.0)	**<**.**001**
Low ACTH	63 (50.0)	14 (23.3)	49 (74.2)	**<**.**001**
Tumor size (cm)	2.4 ± 0.9 (0.8 – 5.0)	2.0 ± 0.6 (0.8 – 3.7)	2.7 ± 1.0 (0.8 – 5.0)	**<**.**001**
Type 2 diabetes mellitus	32 (25.4)	12 (20.0)	20 (15.9)	.221
Osteoporosis	59 (46.8)	19 (31.7)	40 (60.6)	.**001**
Incident VFx	30 (23.8)	6 (10.0)	24 (36.4)	**<**.**001**
LS-BMD (Z-score)	0.10 (−4.10 – 2.50)	0.10 (−2.03 – 3.10)	0.05 (−2.80 – 4.10)	.739
FN-BMD (Z-score)	−0.08 ± 0.86 (−2.4 – 2.7)	0.16 ± 0.76 (−1.6 – 2.1)	−0.01 ± 0.94 (−2.4 – 2.7)	.292

Categorical variables are reported as absolute number with percentage in parentheses. Continuous variables are reported as mean ± SD or median with range in parentheses.

Statistically significant comparisons are in bold.

Abbreviations: ACTH, adrenocorticotrophic hormone; BMD, bone mineral density; BMI, body mass index; F-1mgDST, 1-mg overnight dexamethasone suppression test; FN, femoral neck; Low ACTH, ACTH levels <10.0 pg/mL (2.2 pmol/L); LS, lumbar spine; MACS, mild autonomous cortisol secretion; MACS-no, patients without mild autonomous cortisol secretion (F-1mgDST ≤1.8 µg/dL µg/dL, 50 nmol/L); MACS-yes, patients with mild autonomous cortisol secretion (F-1mgDST >1.8 µg/dL µg/dL, 50 nmol/L); Osteoporosis, BMD T-score < −2.5 at any skeletal site and/or the presence of a fragility fracture; UFC, urinary free cortisol.

**Table 4.** Independent associations between the presence of incident VFx and MACS, age, gender, and LS-BMD by logistic regression analysis

	aRR	95% CI	*P* value
MACS (presence)	4.591	1.585-13.300	**.005**
Age (1-y increase)	1.000	0.944 -1.059	.991
Gender (women)	0.892	0.325-2.444	.824
Type 2 diabetes mellitus (presence)	0.718	0.245-2.103	.546
LS-BMD (1 Z-score decrease)	1.280	0.896-1.829	.174
Patients with prevalent VFx (presence)	1.537	0.598-3.949	.372
Duration of follow-up (1 year increase)	1.003	0.927-1.008	.943

Statistically significant associations are in bold.

Abbreviations: aRR, relative risk adjusted for the variables included in the model; LS-BMD, lumbar spine bone mineral density; MACS, mild autonomous cortisol secretion; VFx, vertebral fragility fracture.

Patients with low ACTH levels showed higher incidence of VFx (31.7%) than patients without low ACTH levels (15.9%, *P* < .036). Finally, the incident VFx were not significantly associated with low ACTH levels after adjusting for age, presence of prevalent VFx and of T2D, gender distribution, duration of follow-up, and LS-BMD.

### Gender-Related Comparisons

**Figure 2.**
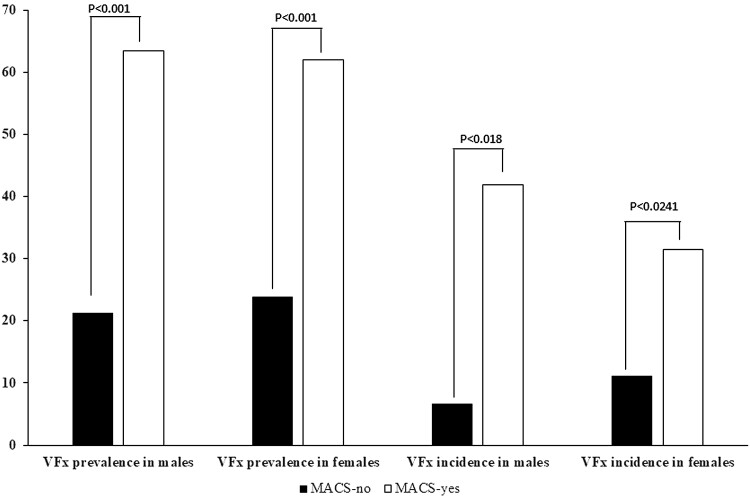
Prevalence and incidence of vertebral fractures in relation with gender and the presence of low bone mineral density in all subjects with adrenal incidentalomas, in patients without mild autonomous cortisol secretion, in patients with possible autonomous cortisol secretion, and in patients with autonomous cortisol secretion. Men and women in the cross-sectional arm: n = 173 and 271, respectively. Men and women in the longitudinal arm: n = 46 and 80, respectively. Abbreviations: MACS-no, patients without mild autonomous cortisol secretion (F-1mgDST ≤1.8 µg/dL µg/dL, 50 nmol/L); MACS-yes, patients with mild autonomous cortisol secretion (F-1mgDST >1.8 µg/dL µg/dL, 50 nmol/L).

The characteristics of men or women without MACS and with MACS included in the cross-sectional arm are reported in Table 5 and the prevalence of VFx in men and women with AI with and without MACS are depicted in Fig. 2. Men with MACS had greater tumor size, and higher age, UFC levels, and prevalence of osteoporosis, VFx, and T2D, while they had lower ACTH levels than men without MACS. Body mass index and BMD did not differ between the 2 groups. In addition, in men with AI, the presence of MACS was associated with prevalent VFx (aOR, 5.23; 95% CI, 2.49-11.00; *P* < .001; aRR 2.62), age (OR 1.054; 95% CI, 1.01-1.09; *P* = .049) and with LS-BMD (OR 1.54; 95% CI; 1.206-1.969; *P* < .001) but not with BMI and presence of T2D. Women with MACS showed higher tumor size and prevalence of osteoporosis and VFx, but lower BMI, ACTH levels, BMD at both LS and FN, and prevalence of premenopausal status than women without MACS. Age, UFC levels, and prevalence of T2D did not differ between the 2 groups. In women with AI the presence of MACS was associated with prevalent VFx (OR 5.36; 95% CI, 3.06-9.38; *P* < .001; aRR 2.21), age (OR 1.06; 95% CI, 1.02-1.10; *P* = .002), and LS-BMD (OR 1.32; 95% CI 1.04-1.67; *P* = .025), but not with BMI, premenopausal status, and presence of T2D.

**Table 5.** Clinical and biochemical characteristics of men or women with adrenal incidentalomas with or without mild autonomous cortisol secretion included in the cross-sectional arm

	Men			Women		
	MACS-no (n = 80)	MACS-yes (n = 93)	*P*	MACS-no (n = 134)	MACS-yes (n = 137)	*P*
Age (years)	58.4 ± 13.1 (21 – 78)	65.8 ± 9.5 (38 – 83)	<.001	60.7 ± 12.9 (26 – 89)	62.1 ± 9.2 (24 – 83)	.318
Premenopausal women	—	—	—	21 (15.7)	10 (7.3)	.**003**
BMI (kg/m^2^)	28.7 ± 3.6 (21.3 – 40.4)	28.6 ± 3.7 (20.3 – 39.0)	.753	30.2 ± 5.6 (19.5 – 40.9)	28.7 ± 521 (19.5 – 40.9)	.**023**
F-1mgDST (µg/dL)	1.15 ± 0.37 (0.50 – 1.80)	3.58 ± 1.84 (1.84 – 9.15)	<.001	1.10 ± 0.40 (0.50 – 1.8)	3.50 ± 2.10 (1.84 – 12.0)	**<**.**001**
UFC (µg/24 hours)	50.6 ± 24.0 (10.0 – 12.0)	61.6 ± 34.8 (10.0 – 170.6)	.019	50.3 ± 29.8 (10.0 – 169.1)	56.6 ± 35.5 (10.0 – 175.3)	.118
ACTH (pg/mL)	18.2 ± 9.6 (5.0 – 48.3)	10.9 ± 6.3 (1.6 – 32.2)	<.001	14.5 ± 9.1 (2.8 – 48.3)	9.9 ± 6.2 (1.6 – 48.0)	**<**.**001**
Tumor size (cm)	2.2 ± 0.9 (0.8 – 6-.0)	3.0 ± 1.1 (1.0-7.0)	<.001	2.1 ± 0.9 (0.8 – 5.5)	3.0 ± 1.2 (0.8 – 8.0)	**<**.**001**
Osteoporosis	27 (33.8)	64 (68.8)	<.001	50 (37.3)	83 (60.6)	**<**.**001**
Type 2 diabetes mellitus	8 (10.0)	23 (24.7)	.**012**	24 (17.9)	18 (13.1)	.278
Prevalent VFx	17 (21.3)	59 (63.4)	**<**.**001**	32 (23.9)	85 (62.0)	**<**.**001**
LS-BMD (Z-score)	−0.06 (−2.80 – 3.61)	−0.10 (−4.5 – 3.61)	.239	.20 (−3.60 – 3.61)	−0.10 (−3.07 – 3.61)	.**006**
FN-BMD (Z-score)	−0.10 (−2.8 – 3.0)	−0.30 (−2.5 – 2.7)	.419	0.10 (−2.6 – 5.3)	−0.20 (−2.5 – 2.5)	.**003**

Categorical variables are reported as absolute number with percentage in parentheses. Continuous variables are reported as mean ± SD or as median with range in parentheses. Statistically significant comparisons are in bold.

Abbreviations: ACTH, adrenocorticotrophic hormone; BMD, bone mineral density; BMI, body mass index; F-1mgDST, 1-mg overnight dexamethasone suppression test; FN, femoral neck; LS, lumbar spine; MACS, mild autonomous cortisol secretion; MACS-no, patients without mild autonomous cortisol secretion (F-1mgDST ≤1.8 µg/dL µg/dL, 50 nmol/L); MACS-yes, patients with mild autonomous cortisol secretion (F-1mgDST >1.8 µg/dL µg/dL, 50 nmol/L); Osteoporosis, BMD T-score < −2.5 at any skeletal site and/or the presence of a fragility fracture; UFC, urinary free cortisol.

The characteristics of men or women without MACS and with MAC included in the longitudinal arm are reported in Table 6 and the incidence of VFx in men and women with AI with and without MACS are shown in Fig. 2. Men with MACS showed older age, higher number of incident VFx, higher UFC levels, larger tumor size, and lower ACTH levels than men without MACS. Duration of follow-up, BMI, prevalence of osteoporosis, prevalent VFx, and presence of T2D did not differ between the 2 groups. In addition, in men the presence of MACS was independently associated with incident VFx (aRR 15.38; 95% CI, 1.25-189.26; *P* = .03) but not with BMD, age, presence of prevalent VFx, and presence of T2D. Women with MACS showed higher number of prevalent and incident VFx, higher prevalence of osteoporosis, larger tumor size, lower ACTH levels, and lower FN-BMD than women without MACS. Age, BMI, duration of follow-up, UFC levels, LS-BMD, premenopausal status, and presence of T2D were comparable. Finally, in women, the presence of MACS was associated with the presence of incident VFx (aRR 3.67; 95% CI, 1.02-13.26; *P* = .047) and of T2D (aRR 4.57; 95% CI, 1.01-20.67; *P* = .048), but not with BMD, age, presence of prevalent VFx, and premenopausal status.

**Table 6.** Clinical and biochemical characteristics of men or women with adrenal incidentalomas with or without mild autonomous cortisol secretion included in the longitudinal arm

	Men			Women		
	MACS-no (n = 15)	MACS-yes (n = 31)	*P*	MACS-no (n = 45)	MACS-yes (n = 35)	*P*
Age (years)	66.0 (45 – 77)	69 (51 – 83)	.**037**	60.6 ± 11.2 (27 – 80)	62.1 ± 7.8 (40 – 73)	.518
Premenopausal women	—	—	—	6 (13.3)	5 (14.3)	.920
BMI (kg/m^2^)	25.8 (23.0 – 32.0)	26.6 (19.0 – 33.1)	.954	27.4 ± 5.0 (20.0 – 40.0)	26.8 ± 4.8 (20.1 – 37.0)	.579
Follow-up (months)	24.0 ± 0.0 (24 – 24)	24.4 ± 2.2 (24 – 36)	.493	24.0 ± 0.0 (24 – 24)	26.7 ± 9.7 (24 – 72)	.061
F-1mgDST (nmol/L)	1.25 (0.50 – 1.80)	3.20 (1.84 – 6.20)	**<**.**001**	1.26 ± 0.41 (0.50 – 1.80)	2.83 ± 1.10 (1.86 – 7.50)	**<**.**001**
UFC (µg/24 hours)	37.8 ± 16.9 (13.8 – 67.5)	58.6 ± 34.4 (10.0 – 150.7)	.**033**	45.8 ± 26.5 (10.0 – 111.9)	51.3 ± 36.6 (10.0 – 175.3)	.446
ACTH (pg/mL)	20.4 ± 10.2 (5.0 – 35.0)	10.0 ± 4.7 (5.0 – 26.7)	**<**.**001**	14.7 ± 11.0 (5.0 – 35.0)	11.0 ± 7.7 (3.0 – 35.0)	.**043**
Tumor size (cm)	1.9 ± 0.8 (0.8 – 3.7)	2.9 ± 1.0 (1.0 – 5.0)	**<**.**001**	2.0 ± 0.6 (0.8 – 3.5)	2.6 ± 1.0 (1.0 – 5.0)	.**02**
Osteoporosis	5 (33.3)	17 (54.8)	.171	14 (31.1)	23 (65.7)	.**002**
Type 2 diabetes mellitus	3 (20.0)	15 (48.4)	.107	9 (20.0)	5 (14.3)	.505
Prevalent VFx	5 (33.3)	19 (61.3)	.075	8 (17.8)	16 (45.7)	.**007**
Incident VFx	1 (6.7)	13 (41.9)	.**018**	5 (11.1)	11 (31.4)	.**024**
LS-BMD (Z-score)	−0.70 (−2.00 – 1.90)	0.50 (−2.20 – 3.50)	.120	.10 (−2.03 – 3.10)	−0.23 (−2.80 – 4.10)	.198
FN-BMD (Z-score)	−0.30 (−1.1 – 1.3)	0.20 (−1.3 – 2.7)	.208	0.30 (−1.6 – 2.1)	−0.20 (−2.4 – 2.0)	.**016**

Categorical variables are reported as absolute number with percentage in parentheses. Continuous variables are reported as mean ± SD or median with range in parentheses.

Statistically significant comparisons are in bold.

Abbreviations: ACTH, adrenocorticotrophic hormone; BMD, bone mineral density; BMI, body mass index; F-1mgDST, 1-mg overnight dexamethasone suppression test; FN, femoral neck; LS, lumbar spine; MACS, mild autonomous cortisol secretion; MACS-no, patients without mild autonomous cortisol secretion (F-1mgDST ≤1.8 µg/dL µg/dL, 50 nmol/L); MACS-yes, patients with mild autonomous cortisol secretion (F-1mgDST >1.8 µg/dL µg/dL, 50 nmol/L); Osteoporosis, BMD T-score < −2.5 at any skeletal site and/or the presence of a fragility fracture; UFC, urinary free cortisol.

## Discussion

The present study shows that in patients with AI and MACS, both prevalence and incidence of VFx are increased compared with patients with AI but without MACS, independently of age, gender distribution, BMI, presence of T2D, and, for incident VFx, even of the presence of a VFx at baseline. The same results were confirmed in men and women when separately evaluated and even after adjusting for premenopausal status in women. In addition, the number of subjects with a prevalent VFx despite a conserved BMD was associated with the degree of cortisol secretion and, in keeping, BMD was not found to be a predictor of incident VFx. Finally, more than one-third of patients with AI and with MACS, and with a conservative approach on follow-up, experienced an incident VFx.

The diagnostic criteria for diagnosing mild hypercortisolism have substantially changed in recent years. Indeed, in the past, several different criteria were used, rendering comparisons very difficult among the studies focused on this condition (3). In 2016, the ESE-ENSAT guidelines suggested that patients with AI should be defined as not affected with MACS, affected with PACS, or with ACS, on the basis of F-1mgDST levels below 1.8 µg/dL (50 nmol/L), between 1.8 µg/dL and 5.0 µg/dL (50-138 nmol/L), or above 5.0 µg/dL (138 nmol/L), respectively (9). Very recently, the updated ESE-ENSAT guidelines suggests that MACS should be diagnosed in patients with AI and with F-1mgDST levels above 1.8 µg/dL (50 nmol/L), without differentiating between ACS and PACS.

This change in the diagnosis of mild hypercortisolism has rendered the previous studies assessing bone health in patients with AI outdated. Indeed, most studies in the past showed that the condition of mild hypercortisolism was associated with increased bone fragility (22-24), and, importantly, some data suggested that the recovery from this condition reduced the risk of incident VFx (6). However, those studies have been conducted by using different criteria for diagnosing mild hypercortisolism as compared with those suggested by the recent ESE-ENSAT guidelines. Indeed, the previous studies did not discriminate between patients with MACS and patients without MACS, since the diagnosis of this mild cortisol excess was generally based on the presence of at least 2 out of 3 altered parameters of HPA axis activity and/or on a F-1mgDST cutoff set at 3 µg/dL (83 nmol/L). The new classification of MACS proposed by the ESE-ENSAT guidelines based on an F-1mgDST cutoff set at 1.8 µg/dL (50 nmol/L) may, therefore, diagnose a larger category of patients with AI as affected with a slight degree of hypercortisolism. Thus, only with the availability of the present data we can assert that the presence of MACS is associated with an increase in the prevalence and/or incidence of VFx. It should be noted, however, that the results of the present study could have been expected on the basis of the independent association between F-1mgDST ≥2.0 µg/dL (55 nmol/L) with the prevalence and incidence of VFx that was found in our previous study (11). However, clear data on bone involvement coming from a specific analysis of patients with MACS compared to those without MACS were not available until now. For this reason, the ESE and ENSAT still consider the association between MACS and osteoporosis not yet well established and suggest that prospective cohorts should evaluate the fracture risk in patients with AI and with MACS (8).

The present study, therefore, is of importance, since it shows on a large sample of individuals that patients with AI and with MACS have an increased prevalence of VFx, confirming previous less homogeneous data. Our results are partially in line with a very recent cross-sectional study by Zavatta and co-authors suggesting that MACS in postmenopausal women with AI was associated with prevalent fragility fractures (10). In addition, we found that even men with MACS have increased prevalence of VFx. This discordance may be explained considering the possible effect played by the difference in disease activity between the 2 studies, since our cohort seemed to have a relatively higher cortisol secretion as compared with the one analyzed by Zavatta et al as mirrored by F-1mgDST levels (present study: mean F-1mgDST 3.6 µg/dL; range, 1.84-9.1 µg/dL vs Zavatta's study: 3.1 µg/dL; range, 2.2-4.2 µg/dL, respectively). In agreement with the importance of the degree of cortisol secretion on the presence of VFx in MACS patients, both the present and Zavatta's studies found an association between F-1mgDST levels with VFx prevalence. Interestingly, we found that even patients with AI, previously defined as affected by PACS, have, in fact, an increased prevalence of VFx as compared with patients without MACS, suggesting that in clinical practice we should not overlook these patients.

Besides the finding of an increased prevalence of VFx in men with MACS, the most important novelty of the present study is that it suggests that even the incidence of VFx is increased in patients with MACS without differences between men and women. This finding has 2 important clinical consequences. First, we should consider that both men and women with MACS are at risk of incident VFx, which, even if asymptomatic, are strong predictors of clinically relevant VFx and hip fracture (20, 25). This is in line with the fact that the last ESE-ENSAT guidelines have decided to suggest screening patients with AI and with MACS for VFx; however, at the time of drafting the guidelines, they considered the relation between MACS and VFx not yet well established (8). Second, if a conservative therapeutical approach is chosen in patients with AI and with MACS, a very careful evaluation of the bone fragility risk should be implemented, since a third of those patients may experience a VFx during the follow-up.

This latter point deserves attention since, to date, a reliable evaluation of the fracture risk in patients with hypercortisolism remains a challenge even for bone experts. Indeed, the fracture risk in patients with hypercortisolism largely depends on the reduction of bone quality rather than on the bone density decrease (26-28). Patients with MACS do not represent an exception. In the present study, indeed, we found that in MACS patients, the VFx prevalence and incidence are increased independently of BMD, with no gender-related differences. Interestingly, we found that the number of patients with fracture and without low BMD increases with the increase of cortisol secretion, suggesting that the higher the cortisol secretion, the more damaged the bone quality is. Unfortunately, nowadays, a reliable assessment of bone quality, particularly in patients with hypercortisolism, is still not obtainable in clinical practice (29, 30). Given the limited role of BMD in evaluating fracture risk in patients with AI and with MACS, the evaluation of other possible risk factors for fracture becomes even more important in MACS patients. In the present study, for example, at least in women, the presence of T2D was associated with the incidence of VFx independently of BMD and the presence of MACS itself. This finding, which was somewhat expected on the basis of the literature (31), suggests that bone fragility should be even more carefully evaluated in women with MACS and T2D. Overall, therefore, although there were no statistically significant differences between the group with MACS and the group without MACS for multiple variables, such as gender distribution, BMI, prevalence of T2D, and premenopausal status, we cannot exclude that cumulatively these variables may contribute to compromised bone health, regardless of the presence of MACS.

This study has some limitations. First, given the retrospective design, we cannot exclude that some possible unknown factors could have biased the results. Second, the relatively small sample size of men included in the longitudinal arm could have prevented the finding of other possible associations. Third, in some patients with AI, cortisol secretion may fluctuate and, thus, measuring the degree of cortisol secretion at the beginning of a follow-up period may not be representative of the disease activity over time in all patients. However, we also found that low ACTH levels were more frequently found in patients with MACS than in those without MACS and that they were associated with the prevalence of VFx. These findings strengthen the idea that the MACS condition has a negative impact on the VFx risk but also that the MACS presence should have been stable over time in our patients. Finally, some confounding factors possibly influencing bone health, such as smoking habit and calcium intake, have not been assessed and some additional data, such as the prevalence and incidence of nonvertebral fractures, which could have provided further information, were not available.

Notwithstanding its limitations, the present study has merit in showing that in both women and men with AI and MACS both the prevalence and incidence of VFx are increased as compared with patients without MACS, independently of BMD and of other possible confounding factors. These results give the support to the last ESE-ENSAT decision to suggest screening of AI patients with MACS for the presence of VFx.

## Data Availability

*Original data generated and analyzed during this study are included in this published article and are available in a data repository at the following link: 10.5281/zenodo.8324621*.

## Abbreviations

ACS: autonomous cortisol secretion
ACTH: adrenocorticotropin
AI: adrenal incidentaloma
aOR: adjusted odds ratio
aRR: adjusted relative risk
BMD: bone mineral density
BMI: body mass index
CT: computed tomography
ENSAT: European Network for the Study of Adrenal Tumors
ESE: European Society of Endocrinology
F-1mgDST: 1-mg-dexamethasone test
FN: femoral neck
HPA: hypothalamic-pituitary-adrenal
LS: lumbar spine
MACS: mild autonomous cortisol secretion
MACS-no: patients without mild autonomous cortisol secretion (F-1mgDST ≤1.8 µg/dL µg/dL 50 nmol/L)
MACS-yes: patients with mild autonomous cortisol secretion (F-1mgDST >1.8 µg/dL µg/dL 50 nmol/L)
PACS: possible autonomous cortisol secretion
T2D: type 2 diabetes
UFC: urinary free cortisol
VFx: vertebral fracture
