# Correction to: “Increased Risk of Vertebral Fractures in Patients With Mild Autonomous Cortisol Secretion”

**DOI:** 10.1210/clinem/dgae246

**Published:** 2024-04-22

In the above-named article by Favero V, Eller-Vainicher C, Morelli V, Cairoli E, Salcuni AS, Scillitani A, Corbetta S, Della Casa S, Muscogiuri G, Persani L, and Chiodini I (*J Clin Endo Metab*. 2024, 109(2), e623-e632; doi: 10.1210/clinem/dgad560), there was an error in the Funding section.

In the originally published article, the Funding section read: “The present work received the following grants: European Union Framework Programme for Research and Innovation —Next Generation EU—NRRP M6C2—Investment 2.1 Enhancement and strengthening of biomedical research in the Italian Ministero della salute, Rome, Italy (PNRR-MAD-2022-12375951) to I.C. and E.C.; RC 2018, RC 2019, RC 2020 to A.S.” In the corrected article, the Funding section reads: “Funded by the European Union - Next Generation EU - NRRP M6C2 - Investment 2.1 Enhancement and strengthening of biomedical research in the NHS.”

The article has been corrected online.

doi: 10.1210/clinem/dgad560

